# Defining the CD39/CD73 Axis in SARS-CoV-2 Infection: The CD73^-^ Phenotype Identifies Polyfunctional Cytotoxic Lymphocytes

**DOI:** 10.3390/cells9081750

**Published:** 2020-07-22

**Authors:** Parimah Ahmadi, Philip Hartjen, Matin Kohsar, Silke Kummer, Stefan Schmiedel, Jan-Hendrik Bockmann, Anahita Fathi, Samuel Huber, Friedrich Haag, Julian Schulze zur Wiesch

**Affiliations:** 1 First Department of Medicine, Section Infectious Diseases, University Medical Center Hamburg-Eppendorf, Martinistr. 52, 20246 Hamburg, Germany; p.ahmadi@uke.de (P.A.); Matin.Kohsar@stud.uke.uni-hamburg.de (M.K.); silkekum@gmail.com (S.K.); s.schmiedel@uke.de (S.S.); j.bockmann@uke.de (J.-H.B.); a.fathi@uke.de (A.F.); s.huber@uke.de (S.H.); 2 Department of Oral and Maxillofacial Surgery, University Medical Center Hamburg-Eppendorf, Martinistr. 52, 20246 Hamburg, Germany; p.hartjen@uke.de; 3 German Center for Infection Research, Hamburg-Lübeck-Borstel-Riems, 23538 Lübeck, Germany; 4 Department of Immunology, University Medical Center Hamburg-Eppendorf, Martinistr. 52, 20246 Hamburg, Germany; haag@uke.de

**Keywords:** COVID-19, purinergic signaling, CD73, CD39, cytotoxic lymphocytes, granzyme B, perforin, SARS-CoV-2, cytokines

## Abstract

The ectonucleotidases CD39 and CD73 regulate immune responses by balancing extracellular ATP and adenosine in inflammation and are likely to be involved in the pathophysiology of COVID-19. Here, we analyzed CD39 and CD73 on different lymphocyte populations in a small cohort of COVID-19 patients and in healthy individuals. We describe a significantly lower level of expression of CD73 on cytotoxic lymphocyte populations, including CD8^+^ T, natural killer T (NKT), and natural killer (NK) cells, during COVID-19. Interestingly, the decrease of CD73 on CD8^+^ T cells and NKT cells correlated with serum ferritin levels. Furthermore, we observed distinct functional differences between the CD73^+^ and CD73^-^ subsets of CD8^+^ T cells and NKT cells with regard to cytokine/toxin secretion. In COVID-19 patients, the majority of the CD73^-^CD8^+^ T cells were capable of secreting granzyme B, perforin, tumor necrosis factor (TNF-α) or interferon-gamma (IFN-γ). To conclude, in this first study of CD39 and CD73 expression of lymphocytes in COVID-19, we show that CD8^+^ T cells and NKT cells lacking CD73 possess a significantly higher cytotoxic effector functionality compared to their CD73^+^ counterparts. Future studies should investigate differences of cellular CD39 and CD73 expression in patients at different disease stages and their potential as prognostic markers or targets for immunomodulatory therapies.

## 1. Introduction

In December 2019, an outbreak of coronavirus disease 2019 (COVID-19), caused by severe acute respiratory syndrome-related coronavirus 2 (SARS-CoV-2) emerged in Wuhan, China [1] and was declared a pandemic by the World Health Organization (WHO) on 11 March 2020.

The main clinical symptoms of COVID-19 include fever, dry cough, and shortness of breath [2]. Approximately 19% of cases are considered severe or critical according to reports of the WHO–China joint mission [3]. These severe cases are characterized by the development of inflammation and pneumonia leading to acute respiratory distress syndrome [4,5]. Current studies suggest that severe courses of infection with SARS-CoV-2 involve immune hyper-activation and an excessive release of cytokines [5], while other studies report an exhausted and dysfunctional immune response [6]. Thus, it has been postulated that clinical treatment strategies that combine the use of antiviral agents/drugs with adequate immunomodulatory therapies are likely to improve clinical outcomes [6,7].

Host factors that influence pro-inflammatory and anti-inflammatory mechanisms play important roles in balancing immunity and immunopathology during viral infections and thus in disease outcome [8]. On a cellular level, an important pathway that regulates lymphocyte activation, homing, and function involves purine nucleotide/nucleoside signaling [9]. ATP released from cells under conditions of stress or by regulated secretion is converted to adenosine (ADO) by the sequential actions of the ectonucleotidases CD39 and CD73. While ATP generally promotes cellular activation and inflammation, adenosine elicits predominantly anti-inflammatory signals [10]. We and others have previously reported dysregulation of CD39 and CD73 on various immune cell populations in viral infections, suggesting a functional role for purine nucleotide/nucleoside signaling in the context of immune responses against viral infections [11,12,13]. In a recent review, M. Abouelkhair discusses the inhibition of ADO signaling by the co-inhibition of CD73 and the ADO receptor Adora2A for the prevention and control of COVID-19 [14].

To better understand the immune responses to SARS-CoV-2 and the particular contribution of the two ectonucleotidases CD39 and CD73 to the immune regulation during COVID-19, we defined two aims for this explorative study. We first focused on the main cellular pathway for the clearance of pathogen-infected cells which is the granule pathway mediated by granzymes and the pore-forming protein perforin. Granzyme B (GrB) and perforin are predominantly expressed and released by cytotoxic T lymphocytes and natural killer cells. We characterized the ex vivo ability of different leukocyte subsets of patients admitted to the hospital with COVID-19 to produce GrB, perforin, tumor necrosis factor (TNF-α) and interferon-gamma (IFN-γ) after short-term ex vivo stimulation with phorbol myristate acetate (PMA)/ionomycin. As our second aim, we sought to investigate the expression profiles of CD39 and CD73 on the same leukocyte subsets to describe potential associations between the expression of the two ectoenzymes and disease severity as well as general functionality and cytotoxic potential. We performed a comprehensive analysis of the ex vivo CD39 and CD73 expression pattern on CD4^+^ T, CD8^+^ T, natural killer T (NKT) and natural killer (NK) cells of COVID-19 patients using a 16-color flow cytometry panel. We compared these findings with observations of samples from uninfected healthy individuals to identify distinct characteristics of the immune responses during SARS-CoV-2 infection.

## 2. Materials and Methods

### 2.1. Study Participants

Fourteen patients diagnosed with COVID-19 infection that were admitted to the infectious diseases ward of the University Medical Center Hamburg-Eppendorf and 12 uninfected healthy individuals were enrolled in this study. Infection with SARS-CoV-2 was tested by qRT-PCR with throat swab samples. Informed, written consent was obtained from all participants. This study was approved by the Ärztekammer Hamburg (PV7298, PV4780). Peripheral blood samples were collected into BD Vacutainer^®^ CPT™ (BD Bioscience, Franklin Lakes, NJ, USA) containing 0.1 M sodium citrate and peripheral blood mononuclear cells (PBMCs) were isolated following the manufacturer’s instruction. Detailed demographic and laboratory patient data are summarized in Table 1.

### 2.2. Intracellular Cytokine Staining

Intracellular cytokine staining was carried out as described elsewhere [15]. Briefly, at least 1 × 10^6^ PBMCs were incubated with PMA (50 ng/mL final concentration) and ionomycin (1 µg/mL final concentration) 1 h before the addition of brefeldin A (1 mg/mL) and monensin (as provided by the supplier). The cells were incubated for 4 h at 37 °C and 5% CO_2_. Unstimulated cells were used as induction control for each sample. PBMCs were then washed and stained with a LIVE/DEAD™ Fixable Near-IR Viability kit (Thermo Fischer, Waltham, MA, USA) to identify live cells as well as the following monoclonal surface antibodies at room temperature for 20 min: BUV395 anti-CD16 (BD Bioscience), BUV737 anti-CD56 (BD Bioscience), BV510 anti-CD20, BV650 anti-CD8, BV711 anti-CD33, PerCp/Cy5.5 anti-CD14, PE/Cy7 anti-CD39, APC anti-CD73, and Alexa Fluor^®^ 700 anti-CD3. Following washing, the PBMCs were fixed and permeabilized using Foxp3/Transcription Factor Staining Buffer Set (eBiosciences™, San Diego, CA, USA) for 45 min at 4 °C. After another washing step, the following intracellular antibodies were added and the cells were incubated for 30 min at 4 °C: BV421 anti-Perforin, BV605 anti-TNF-α, BV785 anti-IFN-γ, FITC anti-CD4, PE anti-IL17, and PE/Dazzle™ anti-Granzyme B. All antibodies were purchased from Biolegend^®^ (San Diego, CA, USA) unless otherwise stated. Finally, the cells were washed and analyzed on a BD LSRFortessa™ (BD Bioscience). A representative gating strategy defining the cell populations analyzed in this study is shown in the Supplementary Figure S9.

### 2.3. Data Evaluation and Statistical Analysis

Analyses of flow cytometry data were performed using FlowJo 10 (Ashland, OR, USA). Statistical analyses were performed using Prism 7.05 software (GraphPad software, San Diego, CA, USA). Gaussian distribution of data was tested using the Kolmogorov–Smirnov test. For intergroup comparisons, student’s t-tests (parametric) and Mann–Whitney tests (non-parametric) were used. For comparison of paired groups, paired student’s t-tests or Wilcoxon tests were used. For bivariate correlation analyses, Pearson’s correlation was performed. Data are presented as mean ± standard deviation (SD). *p* ≤ 0.05 was considered significant. *, **, and *** indicate *p*-values between 0.01 to 0.05, 0.001 to 0.01, and 0.0001 to 0.001, respectively. Analysis and display of complex multivariate flow cytometry data was performed using the software “Simplified Presentation of Incredibly Complex Evaluations” (SPICE) v6.0 [16].

## 3. Results

### 3.1. Characteristics of the Study Participants

In order to functionally characterize lymphocyte subsets during SARS-CoV-2 infection, we collected PBMCs from 14 patients with COVID-19 as well as from 12 healthy uninfected controls. Clinical data from all patients are summarized in Table 1. The patients were 39 to 78 years old (median 54 years), while healthy controls ranged from 25 to 51 years in age (median 30 years). Fifty-seven per cent of patients had relevant comorbidities (cardiovascular/metabolic, respiratory, and oncologic). Five of the patients (36%) needed low-flow nasal oxygen supplementation at some point. Patient C19-09 was later admitted to the ICU and died due to pulmonary embolism (potentially COVID-19-related); all other patients were treated on the regular infectious disease ward. The time from onset of symptoms to PBMC collection was between 5 and 20 days (mean of 11 days). In agreement with published observations [17], COVID-19 patients displayed low or reduced numbers of lymphocytes, including both CD4^+^ and CD8^+^ T cells and B cells (Supplementary Figure S1).

### 3.2. Increased Expression of Granzyme B and Perforin by Cytotoxic Lymphocytes from COVID-19 Patients

**Figure 1:**
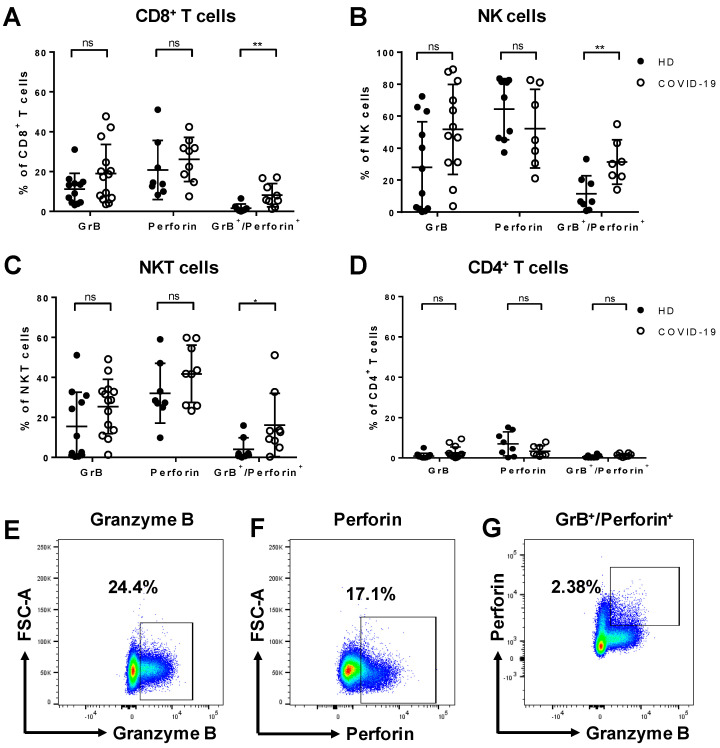
Secretion of granzyme B (GrB) and perforin by different leukocyte populations in COVID-19. Peripheral blood mononuclear cells (PBMCs) of COVID-19 patients and healthy donors (HD) were stimulated ex vivo with phorbol myristate acetate (PMA)/ionomycin for 5 h to analyze the frequency of cytokine-producing cells by flow cytometry. In COVID-19 patients, the frequency of cells co-expressing GrB and perforin was significantly increased among CD8^+^ T cells (**A**) NK cells (**B**), and NKT cells (**C**). The frequency of CD4^+^ T cells secreting GrB or perforin was unaltered upon stimulation (**D**). Representative fluorescence-activated cell sorting (FACS) plots of GrB (**E**), perforin (**F**), and GrB^+^/perforin^+^ (**G**) secretion by CD8^+^ T cells in COVID-19 patients. Data are shown as mean ± SD.

Granzyme B (GrB) and perforin play a key role in the clearance of pathogen-infected cells. Upon stimulation with PMA/ionomycin, we observed an overall rise in the frequency of GrB and/or perforin-secreting cytotoxic lymphocytes in COVID-19 patients compared to healthy donors. The proportion of cells co-producing GrB and perforin was significantly increased among CD8^+^ T cells (*p* = 0.0095, Figure 1A), NK cells (*p* = 0.005, Figure 1B), and NKT cells (*p* = 0.0164, Figure 1C) in COVID-19 patients. Moreover, the median fluorescence intensity (MFI) of GrB was significantly elevated in CD8^+^ T cells (*p* = 0.0142), NK cells (*p* = 0.0036), NKT cells (*p* = 0.0365), and monocytes (*p* = 0.0079) as compared to healthy controls (Supplementary Figure S7). In CD4^+^ T cells, the expression of GrB and perforin was not different from controls (Figure 1D). Representative fluorescence-activated cell sorting (FACS) plots showing the expression of GrB, perforin, or the co-expression of both on CD8^+^ T cells from COVID-19 patients are shown in Figure 1E–G.

Additionally, the proportion of CD8^+^ T cells producing TNF-α was significantly higher in COVID-19 patients compared to healthy controls (*p* = 0.0214), and a similar tendency was observed for CD4^+^ T cells (Supplementary Figure S2).

### 3.3. Expression of CD39 and CD73 by Lymphocyte Subsets from COVID-19 Patients and Healthy Controls

**Figure 2:**
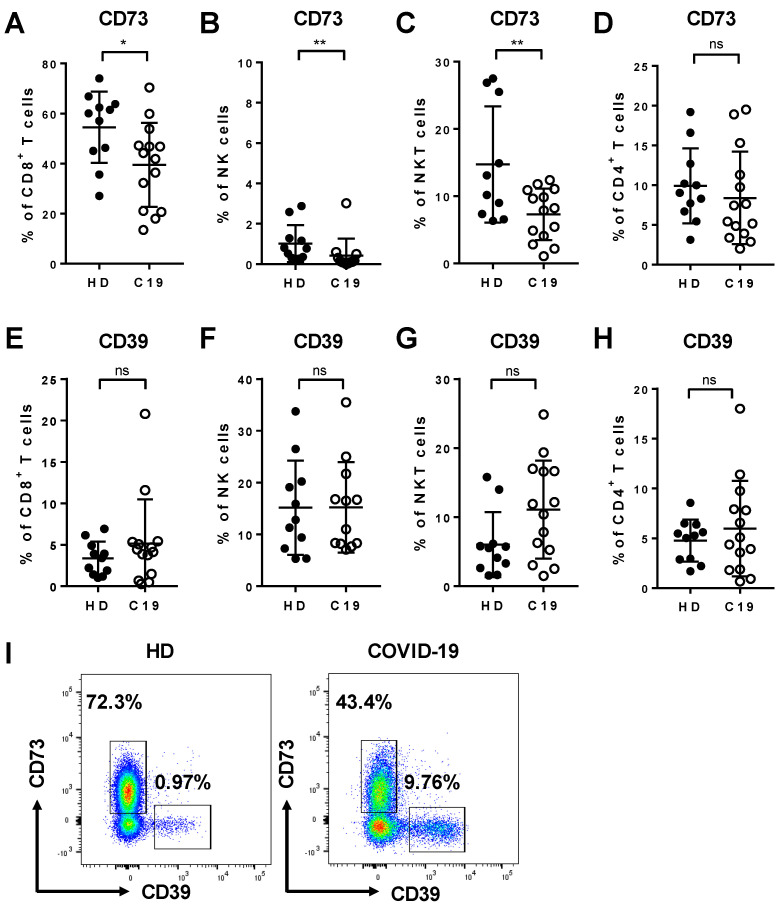
Expression of CD73 and CD39 on different leukocyte populations in COVID-19. PBMCs from COVID-19 patients (C19) and healthy donors (HD) were analyzed ex vivo in unstimulated cells by flow cytometer. In COVID-19 patients, there was a significant decrease in the frequency of CD73^-^expressing CD8^+^ T cells (**A**), NK cells (**B**), and NKT cells (**C**). In contrast, the frequency of cells expressing CD39 was elevated among CD8^+^ T cells (**E**), NK cells (**F**), and NKT cells (**G**) without reaching statistical significance. The expression of both CD73 and CD39 was unaltered on CD4^+^ T cells (**D**, **H**). (**I**) Representative FACS plots of CD39 and CD73 on CD8^+^ T cells in HD and COVID-19. Data are shown as mean ± SD.

We analyzed the expression pattern of the ectonucleotidases CD39 and CD73 on lymphocyte subsets from COVID-19 patients in comparison to healthy controls to characterize their capability to modulate the ATP/adenosine balance. Flow cytometric analysis showed that the frequency of CD73^+^ cells was reduced among CD8^+^ T cells (*p* = 0.0266, Figure 2A), NK cells (*p* = 0.0060, Figure 2B), and NKT cells (*p* = 0.0091, Figure 2C) in COVID-19 patients compared to healthy donors. In contrast, in COVID-19, we observed a tendency towards elevated frequencies of CD39^+^ cells of all three cytotoxic lymphocyte subsets, although these trends did not reach statistical significance, most likely due to the small sample size (Figure 2E–H). We did not observe differences in the expression of CD73 and CD39 on CD4^+^ T cells (Figure 2D,H). However, the median fluorescence intensity of CD73 on all cell populations was reduced in COVID-19 patients in comparison to healthy controls (Supplementary Figure S8). Representative FACS plots showing typical expression levels of CD39 and CD73 on CD8^+^ T cells from healthy donors and COVID-19 patients are shown in Figure 2I.

### 3.4. Lack of CD73 Expression on CD8^+^ T Cells and NKT Cells in COVID-19 Patients Correlates with Clinically-Manifested Systemic Inflammation

**Figure 3:**
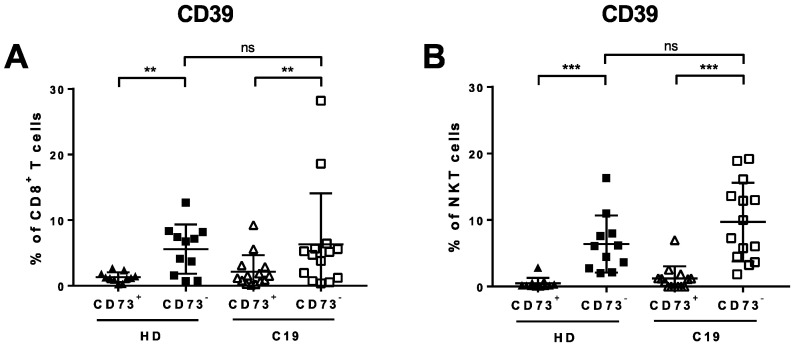
Expression of CD39 on CD73^-^ and CD73^+^ subsets of CD8^+^T cells and NKT cells in COVID-19 (C19) and healthy donors (HD). PBMCs from COVID-19 patients and HD were analyzed ex vivo in unstimulated cells by flow cytometry. CD39^+^ cells were significantly elevated on CD73^-^CD8^+^ T cells (**A**) and CD73^-^ NKT cells (**B**).

We performed a more detailed comparison of the CD73^-^ and CD73^+^ subsets of CD8^+^ T lymphocytes and NKT cells in COVID-19 patients and healthy donors. The frequency of CD39^+^ cells was significantly elevated in both groups within the CD73^-^ populations of both CD8^+^ T cells and NKT cells (*p* = 0.0067 (C19), *p* = 0.0026 (HD), Figure 3A and *p* = 0.0001 (C19), *p* = 0.0010 (HD), Figure 3B, respectively).

**Figure 4:**
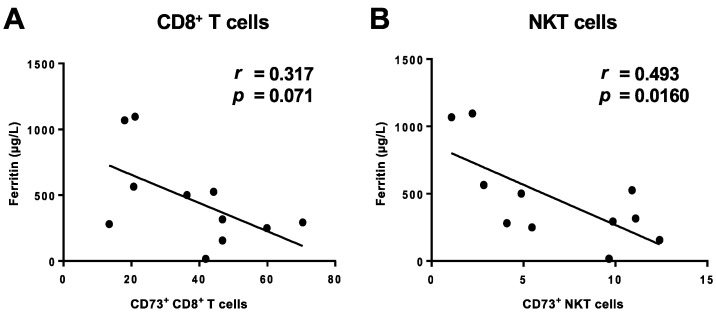
Correlation of CD73 with clinically-manifested inflammation. PBMCs from COVID-19 patients were analyzed ex vivo. Loss of CD73 expression on CD8^+^ T (**A**) and NKT cells (**B**) correlated with the serum ferritin level.

**Figure 5:**
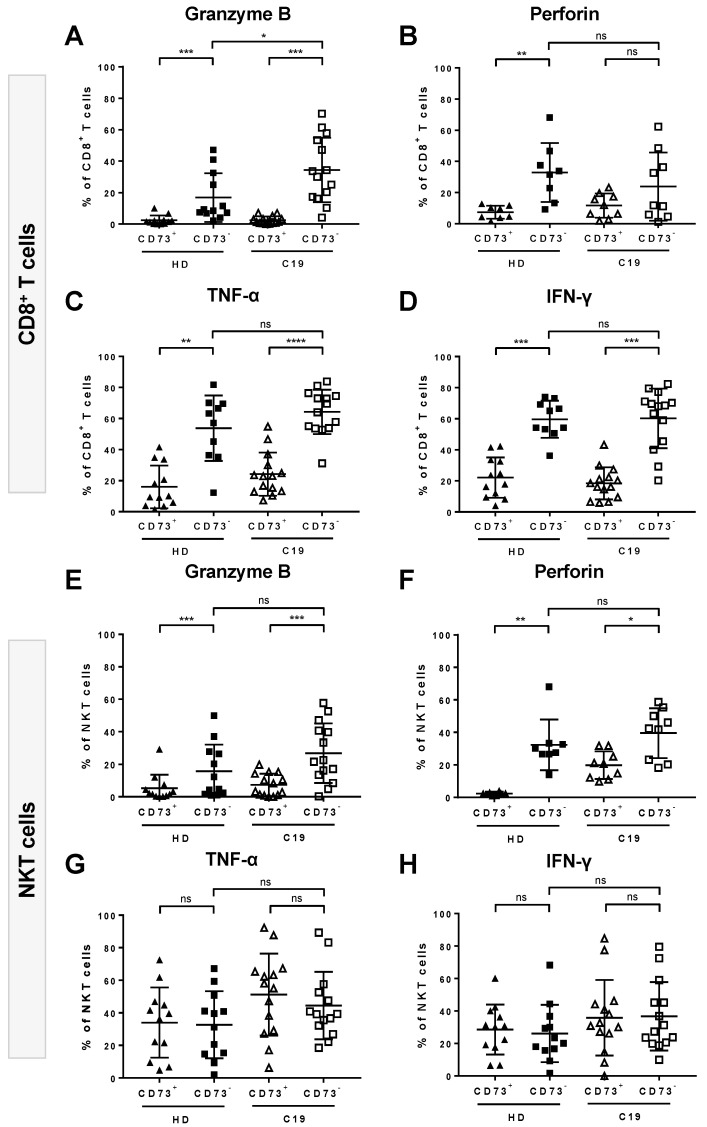
Cytokine/toxin secretion profile of CD73^-^ and CD73^+^ cells among CD8^+^ T and NKT cells. PBMCs from COVID-19 (C19) patients and healthy donors (HD) were analyzed ex vivo upon unspecific stimulation The secretion of granzyme B (GrB) (**A**), TNF-α (**C**), and IFN-γ (**D**) was significantly increased in CD73^-^CD8^+^ T cells compared to their CD73^+^ counterparts in C19 patients and HD. Perforin secretion by CD8^+^ T cells was increased as well, although it only reached a statistical significance for HD (**B**). Regarding NKT cells, GrB (**E**) and perforin (**F**) secretion was significantly elevated in CD73^-^ NKT cells compared to their CD73^+^ counterparts in C19 and HD. TNF-α (**G**) and IFN-γ (**H**) secretion was similar in CD73^-^ and CD73^+^ NKT cells in both C19 and HD. Data are shown as mean ± SD.

We asked whether the loss of CD73 correlated with the degree of systemic inflammation. We therefore correlated the expression levels of CD73 with serum markers of inflammation. We found that the frequencies of CD73^+^ cells in both CD8^+^ T cells and NKT cells fromCOVID-19 patients were negatively correlated with the levels of serum ferritin (Figure 4A,B) while there was no correlation with C-reactive protein (CRP) or interleukin-6 (IL6) (Supplementary Figure S4). Additionally, we compared the cytokine/toxin secretion between CD73^-^ and CD73^+^ cell subsets upon stimulation. We detected significantly elevated GrB (*p* = 0.0001), TNF-α (*p* < 0.0001), and IFN-γ (*p* = 0.0001) secretion by CD73^-^CD8^+^ T cells compared to their CD73^+^ counterparts. Notably, this was the case for COVID-19 patients as well as for healthy donors. While perforin secretion was also increased, it did not reach statistical significance for COVID-19 patients (Figure 5A–D). CD73^-^ NKT cells, on the other hand, secreted significantly higher levels of GrB (*p* = 0.0004 (C19), 0.0005 (HD)) and perforin (*p* = 0.0174 (19), *p* = 0.0078 (HD)) but did not show any differences in TNF-α or IFN-γ secretion compared to CD73^+^ NKT cells (Figure 5E–H).

### 3.5. Absence of CD73 Identifies Highly-Functional Cytotoxic Lymphocytes

**Figure 6:**
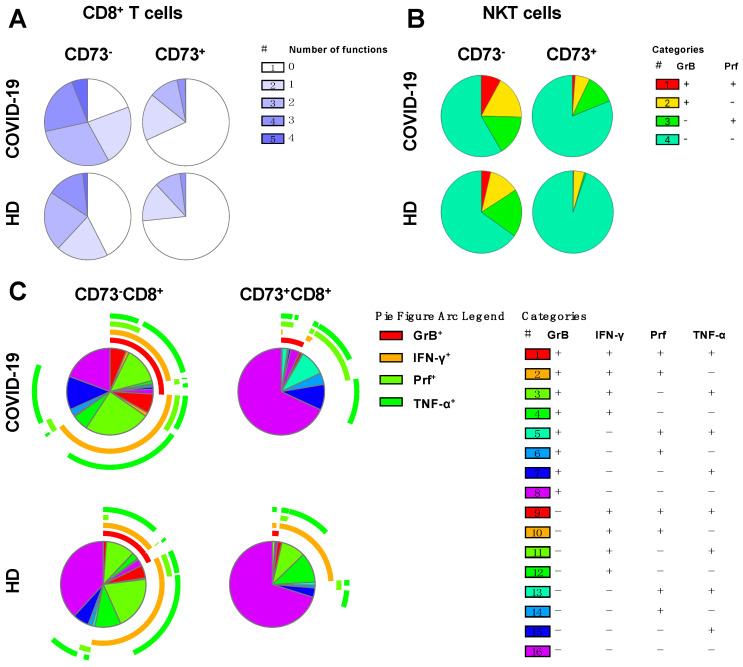
SPICE analysis of TNF-α, IFN-γ, granzyme B (GrB) and perforin (Prf). The pie charts represent the proportions of CD73^-^CD8^+^ T and CD73^+^CD8^+^ T cells with single, double, triple or quadruple functions upon stimulation (**A**). Single or dual (red) secretion of GrB and perforin by CD73^-^NKT and CD73^+^NKT cells upon stimulation (**B**). Different combinations of TNF-α, IFN-γ, GrB and perforin secretion by CD73^-^CD8^+^T and CD73^+^CD8^+^ T cells (**C**).

To investigate the specific ability of CD73^-^ cells to produce single or multiple functional responses, SPICE analyses [16] were performed on CD73^-^CD8^+^ and CD73^+^CD8^+^ T cells in COVID-19 patients and healthy donors. Patterns of singular or combined expression of TNF-α, IFN-γ, GrB, and perforin were examined. Among COVID-19 patients, 81% of CD73^-^ cells produced one or more effector molecules upon stimulation. Of these, 22% produced three and 6% produced four effector molecules. By contrast, only 31% of CD73^+^ cells were positive for one or more effector molecules with 28% secreting only one or two molecules and 3% producing three molecules (Figure 6A).

Similarly, in healthy donors, CD73^-^CD8^+^ T cells were also more functional than their CD73^+^ counterparts. However, in general their functionality was lower than that of cells from COVID-19 patients. Only 62% of cells (compared to 81% in COVID-19) produced any effector molecule, and the proportion of the cell population capable of producing all four functional molecules was on average three times smaller than in COVID-19 patients (2% in HD vs. 6% in C19, Figure 6A). The distribution of CD73^-^CD8^+^ or CD73^+^CD8^+^ T cell populations producing any combination of TNF-α, IFN-γ, GrB, or perforin is depicted in Figure 6C.

CD73^-^ NKT cells secreted significantly higher amounts of GrB and perforin upon stimulation compared to their CD73^-^ counterparts, but this was not the case for TNF-α or IFN-γ (Figure 5E–H). Therefore, we compared the CD73^-^ and CD73^+^ subpopulations concerning their ability to co-secrete the two toxins. We found that 8% of CD73^-^ NKT cells produced both GrB and perforin compared to 2% in CD73^+^ NKT cells. As observed in CD8^+^ T cells, CD73^-^ NKT cells from COVID-19 patients exhibited a greater degree of functionality than the corresponding population from healthy donors (Figure 6B).

**Figure 7:**
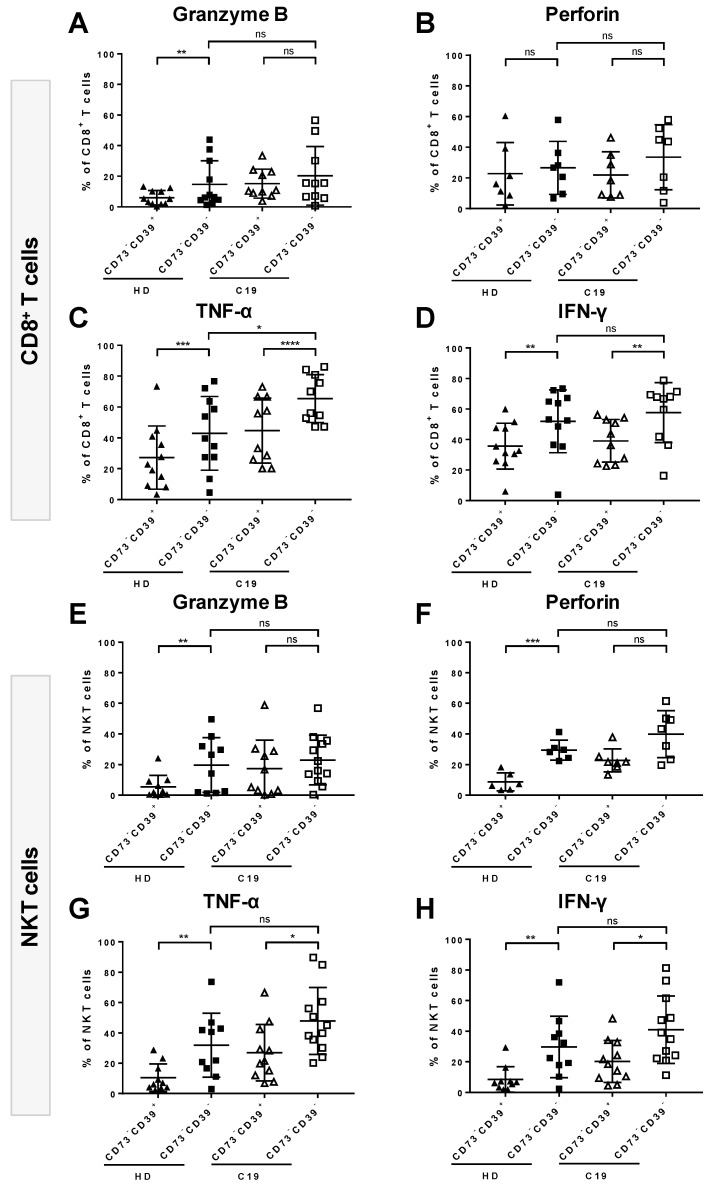
Comparison between the CD39^+^CD73^-^ and CD39^-^CD73^-^ cell subsets for the ability to secrete granzyme B (GrB), perforin, TNF-α and IFN-γ in COVID-19 (C19) and healthy donors (HD). PBMCs of C19 patients and HD were analyzed upon unspecific stimulation. Within CD8^+^ T cells in C19, the secretion of TNF-α (**C**) and IFN-γ (**D**) was significantly increased in CD73^-^CD39^-^ T cells compared to their CD73^-^CD39^+^ counterparts. In HD, in addition to TNF-α (**C**) and IFN-γ (**D**), the proportion of GrB (**A**) secreting cells was significantly elevated among CD73^-^CD39^-^ cells. Within NKT cells in C19, GrB (**E**) and perforin (**F**) were moderately increased while the secretion of TNFα (**G**) and IFN-γ (**H**) was significantly elevated on the CD73^-^CD39^-^ subset compared to the CD73^-^CD39^+^ subset. In HD, the secretion of all four tested functional molecules was elevated among CD73^-^CD39^-^ T cells. Data are shown as mean ± SD.

Intriguingly, within the CD73^-^ cell compartment of both CD8^+^ T cells and NKT cells, cytokine- or toxin-secreting cells tended to be enriched even more in the CD39^-^ subset in both COVID-19 patients and healthy donors. (Figure 7). The frequency of TNF-α- and IFN-γ-secreting cells was significantly higher within the CD73^-^CD39^-^ than in the CD73^-^CD39^+^ cell subset for both CD8^+^ T cells (*p* = <0.0001, Figure 7C, *p* = 0.0059, Figure 7D, respectively) and NKT cells (*p* = 0.0196 Figure 7G, *p* = 0.0276, Figure 7H, respectively). Similarly, the proportions of GrB- and perforin-producing cells appeared to be increased among CD73^-^CD39^-^ cells, although this effect did not reach statistical significance (Figure 7A,B,E,F). In comparison, in healthy donors, the secretion of all tested cytokines/toxins was significantly increased among CD73^-^CD39^-^ cells in both CD8^+^ T cells and NKT cells, with the exception of perforin produced by the CD73^-^CD39^-^ subset of CD8^+^ T cells.

Since the frequency of CD73^+^ NK cells was very low in COVID-19 patients (mean 0.433%) as well as healthy donors (mean 1.0%, Figure 2B), a comprehensive comparison between CD73^+^ and CD73^-^ NK cell subpopulations was not possible.

## 4. Discussion

The ectoenzymes CD73 and CD39 can hydrolyze exogenous ATP to adenosine, thereby attenuating inflammation [18]. We show the expansion of a highly-functional subset of cytotoxic lymphocytes during infection with SARS-CoV-2 that was characterized by the absence of CD73 on the cell surface. Overall, we saw a markedly altered presence of both ectoenzymes on the surface of cytotoxic lymphocytes. Moreover, we examined the functional differences between CD73 deficient CD8^+^ T cells and NKT cells and their CD73^+^ counterparts regarding their cytokine/toxin secretion profile. CD73^-^ cells were generally more capable of secreting GrB, perforin, TNF-α, or IFN-γ regardless of the disease status, but this effect was stronger in lymphocytes obtained from COVID-19 patients compared to those obtained from healthy donors (Figure 6A). In CD4^+^ T cells (Figure 2D) and monocytes (data not shown) the surface expression of CD39 and CD73 was not altered, pointing towards a specific effect in cytotoxic lymphocytes, including CD8^+^ T cells, NK cells, and NKT cells.

Despite their complementary function, CD39 and CD73 are rarely co-expressed on peripheral human lymphocytes (with the exception of B cells) [19]. In humans, CD73 is generally expressed on endothelial cells, B cells, naïve CD8^+^ T cells, and a subset of memory CD4^+^ T cells [20]. Recent studies show that upon activation, CD73 may be shed from the cell surface resulting in a disturbance of ATP/ADO balance in the extracellular matrix (ECM) [20,21]. In fact, cells that lose CD73 could be protected by pericellular ADO, thus maintaining their effector function [20]. A specific mechanism for the observed loss of CD73 on cytotoxic lymphocytes in COVID-19 remains to be elucidated. It could reflect the shedding of CD73 from the surface of CD8^+^ T cells following activation or may alternatively be the consequence of a reduction in the frequency of naïve CD8^+^ T cells. We analyzed the activation and exhaustion status of T cells from 10 of the COVID-19 patients in a separate project. Although we did not measure the expression of CD73 on individual subpopulations in these experiments, the data could be used to investigate a possible correlation between surface CD73 expression and the differentiation or activation status of CD8^+^ T cells. In this small series, we did not observe any correlation between CD73 expression and the differentiation (CCR7, CD45RO, and CD27) or activation status (HLA-DR and CD38) of CD8^+^ T cells.

Our first aim was to study alterations in the expression of components of the granule pathway for the clearance of pathogen-infected cells which is mediated by granzymes and the pore-forming protein perforin. Despite the findings of Zheng et al., we did not observe functional exhaustion of T cells in SARS-CoV-2-infected cells in our study cohort [6]. Instead, we demonstrate an increase in the frequency of GrB and perforin-co-secreting cytotoxic lymphocytes (Figure 1) and elevated GrB MFI in all tested leukocyte subsets (Supplementary Figure S7) upon unspecific stimulation with PMA/ionomycin. Moreover, we found a rise in TNF-α-secreting CD8^+^ T cells as well as a similar tendency in CD4^+^ T cells (Supplementary Figure S2) obtained from COVID-19 patients. GrB is a serine protease that is dominantly secreted by NK cells and cytotoxic T lymphocytes. Upon secretion, it enters the cytoplasm of infected cells mainly through the formation of pores by perforin, and induces apoptosis [22]. TNF-α is a pro-inflammatory cytokine that also promotes apoptosis, in this case by binding to the TNFR1 receptor on T cells. Previous studies have reported the contribution of TNF-α to the cytokine storm syndrome in more severe cases of COVID-19 [23]. The increased ability of CD8^+^ T cells derived from patients in our study cohort to secrete TNF-α upon stimulation could potentially be involved in the cytokine storm process, although we have not performed in-depth analyses to test this hypothesis. In contrast to the cytotoxic lymphocytes, CD4^+^ T cells displayed no or only very limited levels of GrB, perforin, TNF-α, or IFN-γ secretion in COVID-19 patients (Figure 1D and Supplementary Figure S2), in line with the distinct role of cytotoxic lymphocytes for the clearance of pathogen-infected cells through the granule pathway that differs from the canonical role of CD4^+^ T cells. The described apoptosis signaling pathways could potentially play a role in the decrease of the total lymphocyte numbers in our cohort of COVID-19 patients (Supplementary Figure S1). These data confirm previous findings reporting that 75% of COVID-19 patients had lymphopenia [17]. We also analyzed the frequency of cytokines/toxins in unstimulated samples. While the expression of TNF-α and IFN-γ in unstimulated samples was typically below 1%, GrB and perforin showed similar expression levels in stimulated and unstimulated samples. A comparison is depicted in Supplementary Figure S6.

Our second aim was to assess distinct characteristics of lymphocyte subsets regarding the surface expression pattern of the two ectonucleotidases CD39 and CD73 in SARS-CoV-2 infection. Under conditions of stress, as during an infection or upon cell damage, ATP is released by cells and acts on the ECM-stimulating P2 receptor, generally inducing pro-inflammatory signals. CD73 and CD39 act complementarily in the degradation of ATP and the production of adenosine (ADO), which binds to P1 receptors on immune cells, predominantly eliciting anti-inflammatory signals. CD73, which is the rate-limiting enzyme for the production of ADO, plays a key role in the control of inflammatory signals [9]. A functional role for CD73 in regulating immune response is known in the tumor microenvironment, where it contributes to the reduction of anti-tumor immune responses [24]. Further support for the pathophysiological role of CD73 is shown in our previous works with chronic viral infections such as HIV infection, where CD73 deficiency of CD8^+^ T cells was associated with chronic immune activation [13]. Also, Schuler et al. have shown the contribution of CD4^+^CD73^+^ T cells in the suppression of cytokine secretion in HIV infection through ADO production. Together these data suggest that the depletion of ADO-producing CD4^+^ T cells in HIV infected patients could contribute to increased general immune activation in these patients [25].

Recently, diabetes mellitus types 1 and 2 have been reported as risk factors for disease progression and poor prognosis of COVID-19 [26,27]. Interestingly, a protective role of CD73 has been shown in murine diabetes models. In these mice, CD73 levels in the kidney were elevated and genetic deletion of CD73 or the gene encoding ADO receptor, *Adora2b*, resulted in a more severe diabetic nephropathy [28]. Additionally, in murine TLR9-/- models it has been shown that an elevated expression of CD73 on immune cells was associated with the production of anti-inflammatory cytokines by CD4^+^ T cells and delayed diabetes development [29]. Based on these findings, it could be postulated that alterations in the CD39/CD73 axis and the loss of CD73 in cytotoxic lymphocytes in COVID-19 could lead to a lower ADO concentration in the ECM, which could contribute to the faster progression and worse outcome of both diabetes and COVID-19. Also, it remains to be tested whether these CD73^-^ cytotoxic lymphocytes become highly activated in the presence of ATP due to the lack of an ability to produce ADO which would bind to the inhibitory ADO receptors in an autocrine manner [30].

Interestingly, we could show that CD73^-^ cells that expressed CD39 on their surface, tended to be less functional with regards to cytokine secretion compared to CD73^-^CD39^-^ cells within CD8^+^ T and NKT cells. Our data show that for both CD8^+^ T and NKT cells, the restriction of cytokine and toxin production to the CD73^-^CD39^-^ subsets is a property common to both COVID-19 patients and healthy donors (Figure 7). We found that the CD73^-^ subset as a whole was expanded among CD8^+^ T cells and NKT cells from COVID-19 patients compared to healthy donors (Figure 2), while within this subpopulation, the division into CD39^+^ and CD39^-^ did not differ significantly between COVID-19 patients and healthy donors (Supplementary Figure S5). In summary, we interpret the data as showing that COVID-19 patients possess an elevated frequency of CD73^-^ cells and that within this cell population, effector functions such as cytokine and toxin secretion are largely restricted to the CD39^-^ subpopulation.

Intriguingly, the loss of CD73 correlated with plasma ferritin levels, underscoring a potential role of ferritin as a prognostic marker for inflammation in COVID-19. Indeed, patient C19-09, who was later admitted to the ICU and died due to pulmonary embolism, had one of the highest plasma ferritin levels (1068 [µg/L]). As an acute-phase protein, elevated serum ferritin levels are common in infectious diseases and non-infectious inflammatory states. Serum ferritin levels are particularly elevated in various viral infections, partly to extreme ranges of more than 1000 µg/L, while CRP and IL-6 values are often only mildly elevated [31]. The incidence of high values of serum ferritin in COVID-19 patients, as observed in our cohort, points towards the severe inflammatory state induced by SARS-CoV-2 and the pro-inflammatory pathways induced by ferritin potentially predominate in COVID-19.

In this study, we examined the cytokine/toxin secretion ability of different lymphocyte subsets upon an unspecific stimulation with PMA/ionomycin. Thus, we cannot discriminate whether our observations with regards to cytokine/toxin secretion by different lymphocyte subsets are directly induced by antigen exposure or by an inflammatory bystander activation. Moreover, our study is limited by the relatively small cohort of SARS-CoV-2 infected patients that did not allow for comparisons between subgroups with differential disease outcomes and was statistically underpowered for more detailed sub-analyses. On average, the control group of healthy volunteers was younger than the group of COVID-19 patients, but we did not observe any correlation between the age of donors and CD73 expression.

Taken together, we describe a subset of poly-functional cytotoxic lymphocytes in COVID-19 characterized by remarkably lowered CD73 expression. Further studies are required to assess the involvement of these cells in the development of antigen-specific responses during the course of COVID-19 disease. Moreover, comparisons between severe and mild cases would provide insights towards a prognostic value of the absence of CD73 in immune activation and disease.

## Figures and Tables

**Table 1:** Demographic and laboratory patient information.

Patient	Days Since Symptom Onset	Age	Sex	Relevant Pre-Existing Medical Conditions	Relevant Medication	CRP [mg/L]	IL-6 [ng/L]	Serum Ferritin [µg/L]	Oxygen Demand *	Lowest SpO_2_ [%]
C19-01	9	61	m	None	Ampicillin/sulbactam, Piperacillin/tazobactam, Levofloxacin	58	n/a	n/a	–	89
C19-02	8	51	m	None	None	53	40.2	501	–	95
C19-03	6	78	m	COPD GOLD 2B, Coronary heart disease, hypertension	Ampicillin/sulbactam	97	63.3	1096	2 L/min	82
C19-04	13	58	m	None	Ampicillin/sulbactam, Piperacillin/tazobactam, meropenem	21	21	1140	6 L/min	75
C19-05	5	58	m	None	None	108	38.3	316	–	95
C19-06	12	49	m	NSCLC, Hodgkin’s disease 1991 (CR)	Hydroxychloroquine, azithromycin, cisplatin, paclitaxel, pembrolizumab (03/2020)	33	9.1	155	3 L/min	93
C19-07	10	75	m	Myocardial infarction (2004), urothelial carcinoma	None	56	335	564	–	95
C19-08	12	50	m	Asthma	None	12	1.9	280	2 L/min	95
C19-09	11	63	m	Asthma, type 2 diabetes	None	133	125	1068	4 L/min	84
C19-10	9	46	m	Hypertension	None	8	5.4	n/a	–	94
C19-11	20	39	m	None	Ampicillin/sulbactam	46	4.7	249	–	94
C19-12	20	53	m	Type 2 diabetes	None	7	1.5	526	–	–
C19-13	14	44	w	Multiple sclerosis, Breast cancer (2010)	Rituximab (last dose: 01/2020)	33	371.9	292,7	–	94
C19-14	10	56	w	None	Hydroxychloroquine	38	19.9	16,7	–	93

Abbreviations: COPD: chronic obstructive pulmonary disease; CRP: C-reactive protein; IL-6: interleukin-6; NSCLC: non-small-cell lung carcinoma. * nasal oxygen supply.

## Supplementary Materials

The following are available online at https://www.mdpi.com/2073-4409/9/8/1750/s1 Figure S1: Decreased cell numbers within leukocyte subsets, Figure S2: Secretion of TNF-α and IFN-γ by different leukocyte subsets in COVID-19 patients and healthy donors, Figure S3: Secretion of Granzyme B, perforin, TNF-α and IFN-γ by monocytes, Figure S4: Correlation of CD73 surface expression on CD8^+^ T cells and NKT cells with CRP and IL-6, Figure S5: CD39 surface expression on CD73^-^CD8^+^ T cells and CD73^-^NKT cells, Figure S6: Secretion of Granzyme B and perforin by unstimulated CD73^-^CD8^+^ T and CD73^-^ NKT cells and their CD73^+^ counterparts in COVID-19 patients and healthy donors, Figure S7: Median fluorescence intensity of Granzyme B, perforin, TNF-α and IFN-γ in CD8^+^ T cells, NK cells, NKT cells and monocytes, Figure S8: Median fluorescence intensity of CD39 and CD73 on different lymphocyte subsets, Figure S9: Gating strategy to define NK cells, NKT cells and CD4^+^/CD8^+^ T cells.
