# Supplementary material for: Defining the CD39/CD73 Axis in SARS-CoV-2 Infection: The CD73- Phenotype Identifies Polyfunctional Cytotoxic Lymphocytes
Source: Cells. 2020 Jul 22;9(8):1750. doi: 10.3390/cells9081750 (PMC7464076; doi:10.3390/cells9081750)
Supplement: Supplementary file 1 [file cells-09-01750-s001.pdf]

cells

Supplementary Material

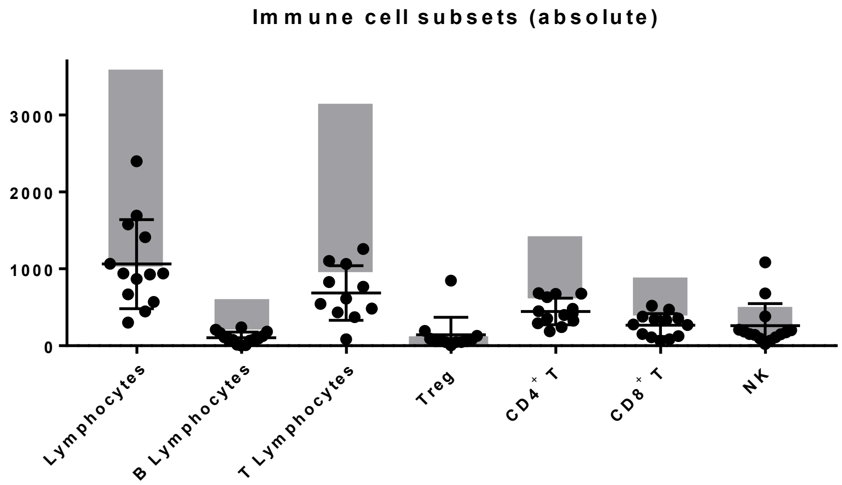

Supplementary Figure S1: Decreased cell numbers within leukocyte subsets. PBMC from COVID-19 patients were analyzed ex vivo by flow cytometry. Reference ranges of individual leukocyte subsets for healthy donors are shown in grey. Data are shown as mean ± SD.

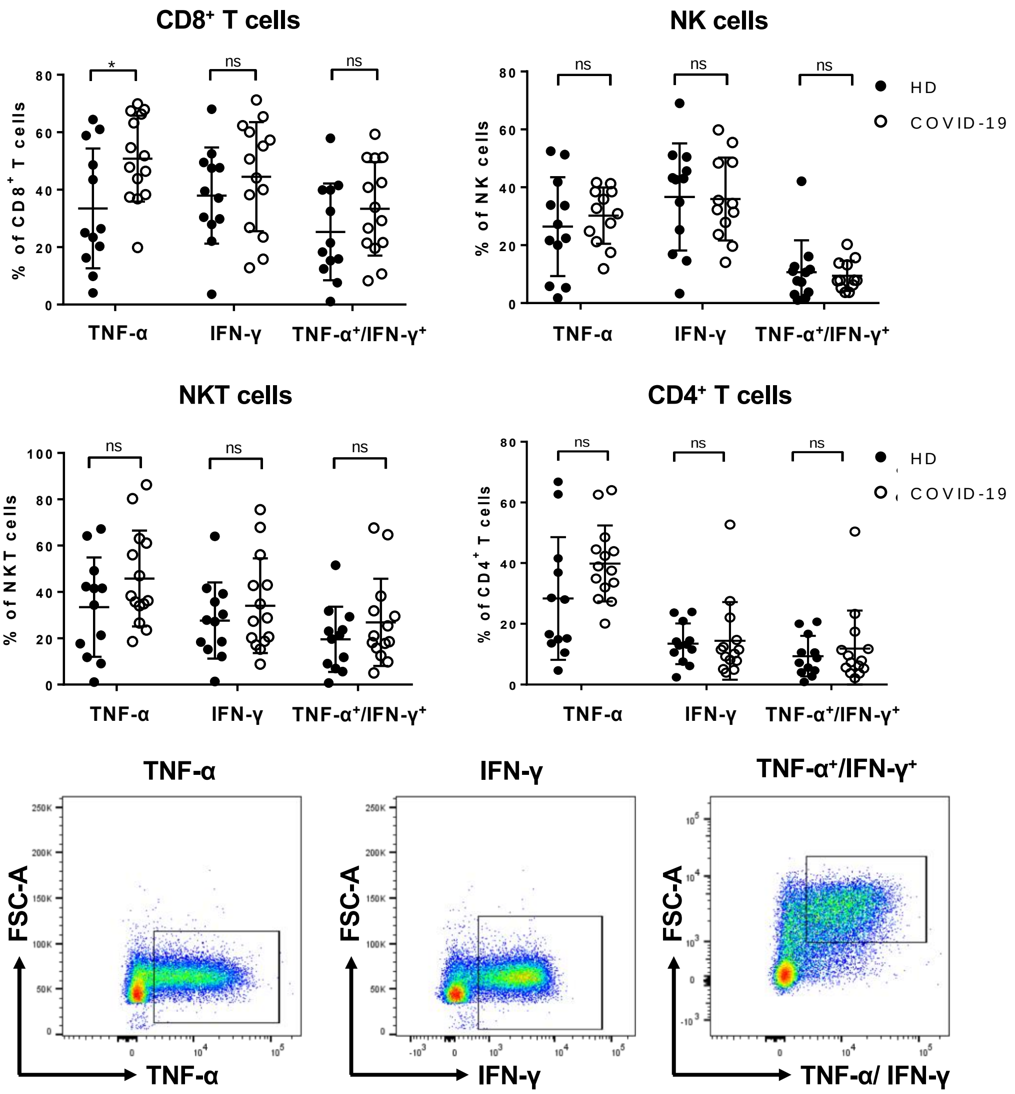

Supplementary Figure S2: Secretion of TNF-α and IFN-γ by different leukocyte subsets in COVID-19 patients and healthy donors (HD). PBMC from COVID-19 patients and HD were stimulated ex vivo with PMA/ionomycin for 5h to analyze the frequency of cytokine producing cells by flow cytometry. Dot plots represent CD8⁺ T cells from COVID-19 patients. The frequency of cytokine-producing cells in unstimulated samples was typically below 1%. Data are shown as mean ± SD.

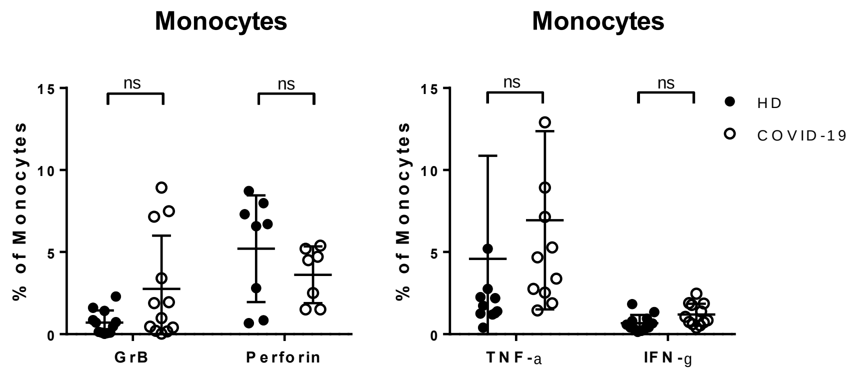

Supplementary Figure S3: Secretion of Granzyme B (GrB), perforin, TNF-α and IFN-γ by monocytes. PBMC from COVID-19 patients and healthy donors (HD) were stimulated ex vivo with PMA/ionomycin for 5h. The frequency of cytokine-producing cells among monocytes was analyzed by flow cytometry. Data are shown as mean ± SD.

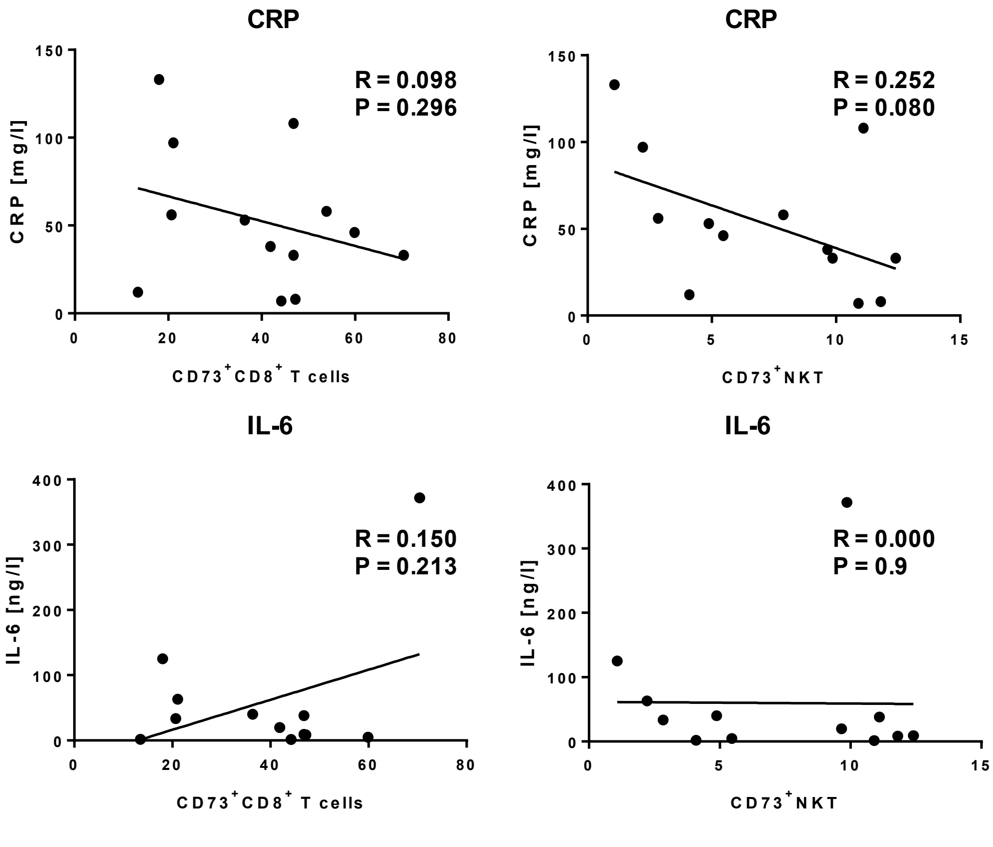

Supplementary Figure S4: Correlation of CD73 surface expression on CD8+ T cells and NKT cells with CRP and IL-6.

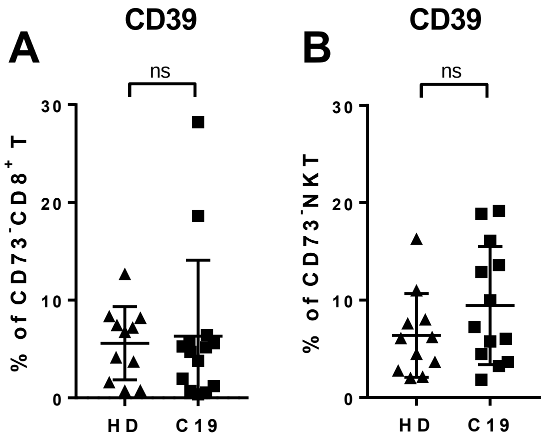

Supplementary Figure S5: CD39 surface expression on $CD73^{-}CD8^{+}$ T cells and $CD73^{-}$NKT cells. PBMCs from healthy donors (HD) and COVID-19 (C19) patients were analyzed ex vivo by flow cytometry and compared in regard to CD39 surface expression on $CD73^{-}CD8^{+}$ T cells **(A)** and $CD73^{-}$ NKT cells **(B)**. Data are shown as mean ± SD.

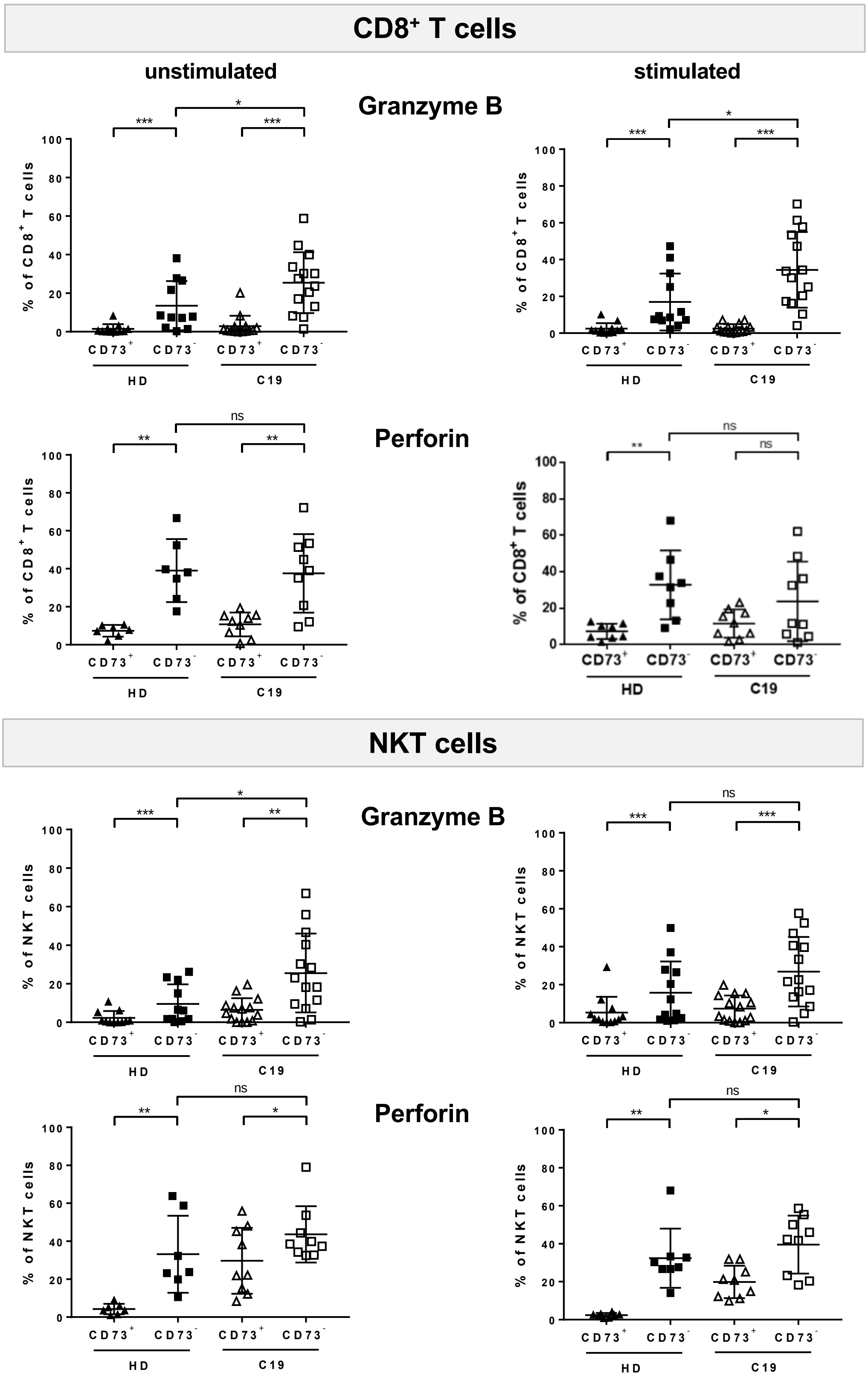

Supplementary Figure S6: Secretion of Granzyme B and perforin by unstimulated CD73⁻CD8⁺ T and CD73⁻NKT cells and their CD73⁺ counterparts in COVID-19 (C19) patients and healthy donors (HD). Data are shown as mean ± SD.

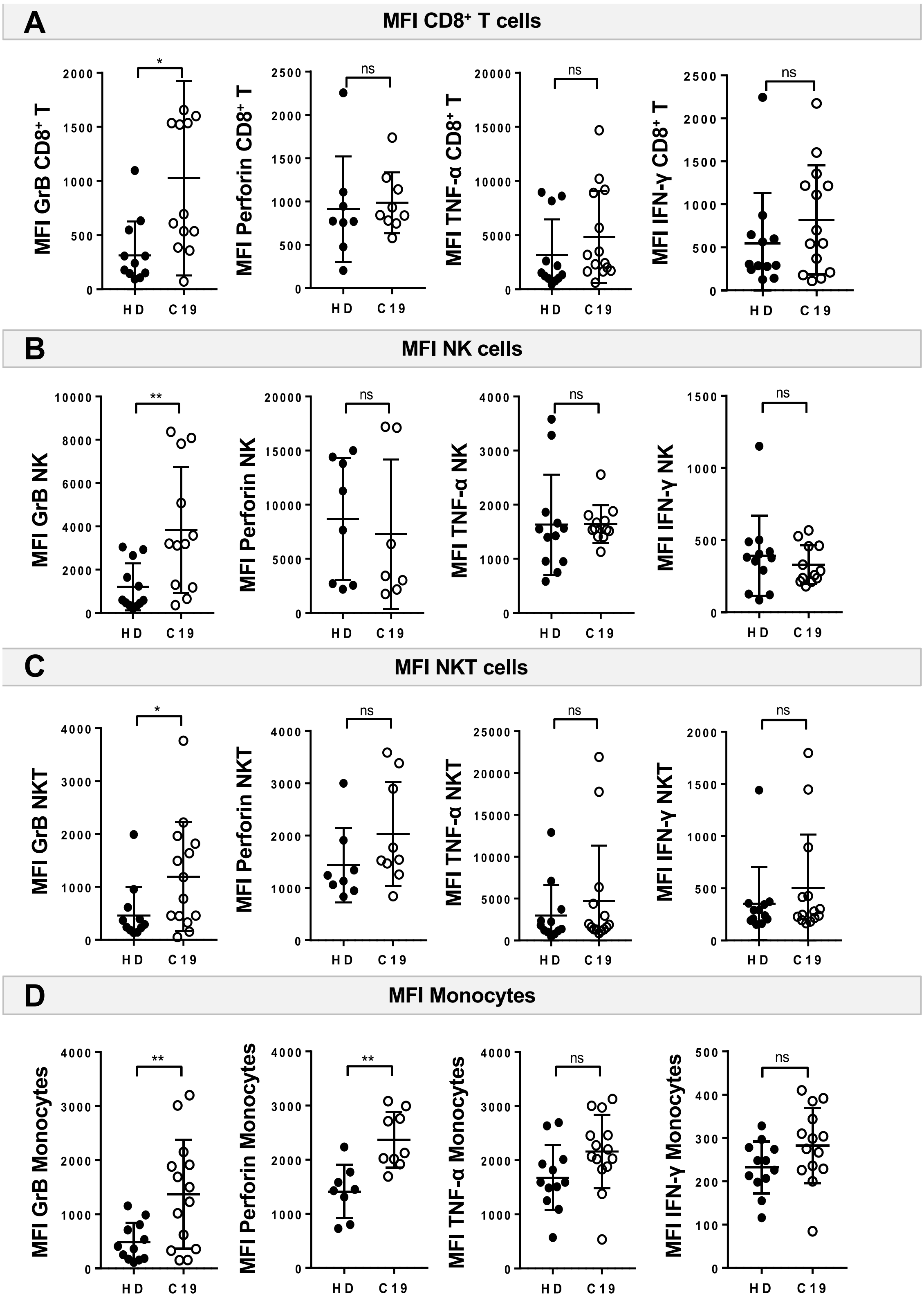

Supplementary Figure S7: Median fluorescence intensity (MFI) of Granzyme B (GrB), perforin, TNF-α and IFN-γ in CD8⁺ T cells (A), NK cells (B), NKT cells (C) and monocytes (D) in COVID-19 (C19) patients and healthy donors (HD). Data are shown as mean ± SD.

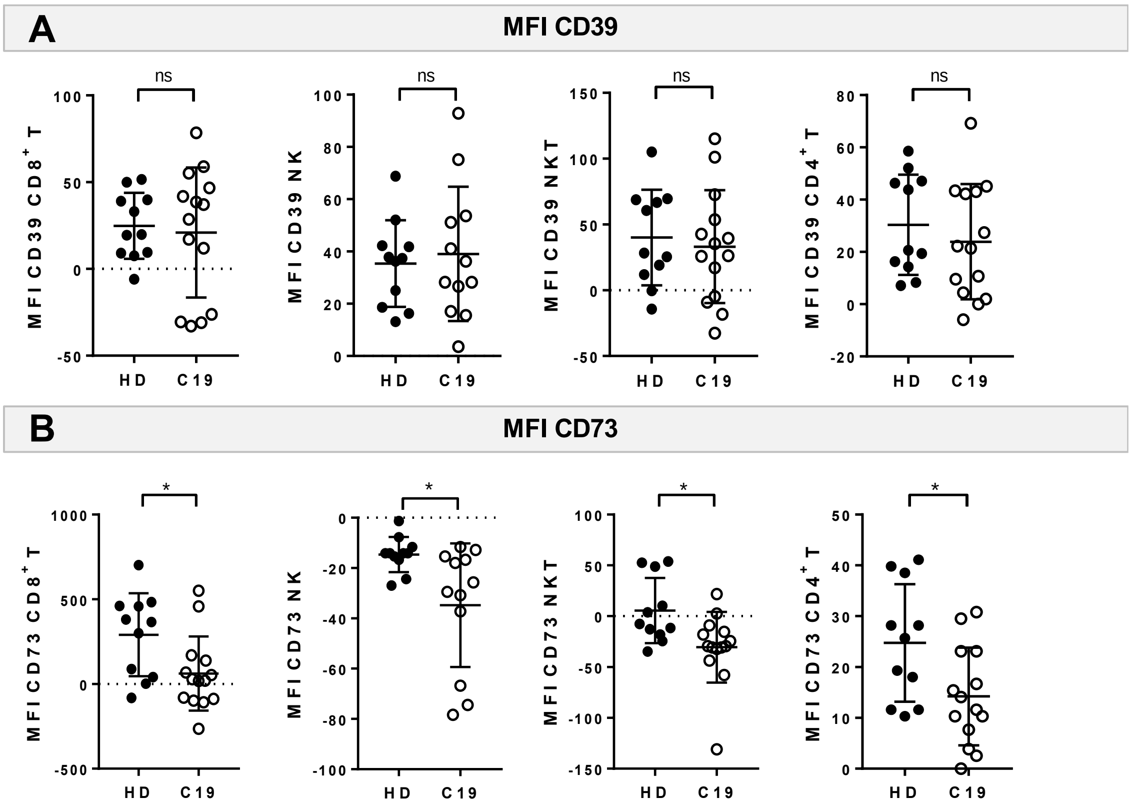

Supplementary Figure S8: Median fluorescence intensity (MFI) of CD39 and CD73 on different lymphocyte subsets. PBMC from COVID-19 (C19) patients and healthy donors (HD) were analyzed ex vivo by flow cytometry. Data are shown as mean ± SD.

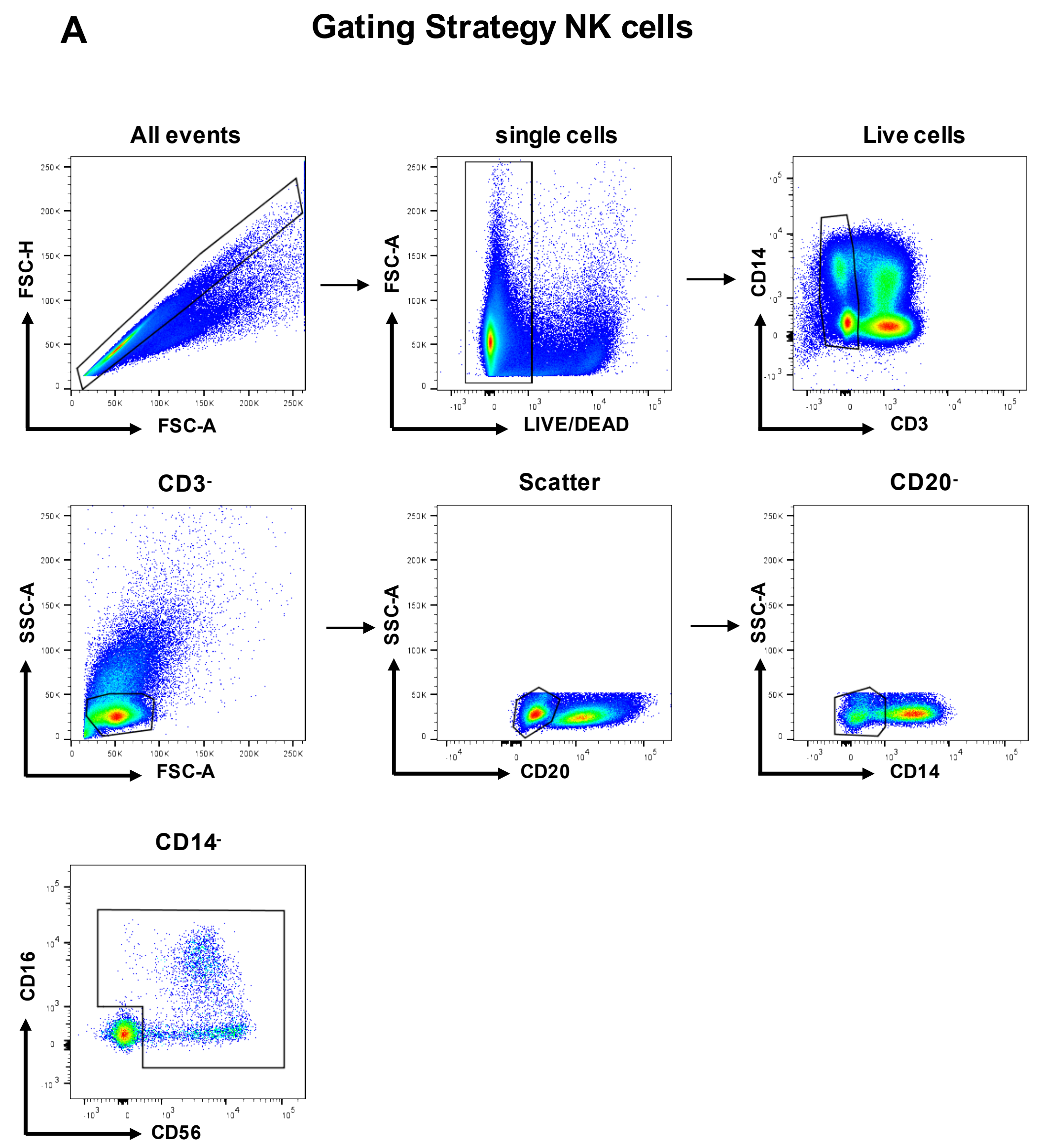
A
Gating Strategy NK cells
All events
FSC-H
FSC-A
single cells
FSC-A
LIVE/DEAD
Live cells
CD14
CD3
CD3-
SSC-A
FSC-A
Scatter
SSC-A
CD20
CD20-
SSC-A
CD14
CD14-
CD16
CD56

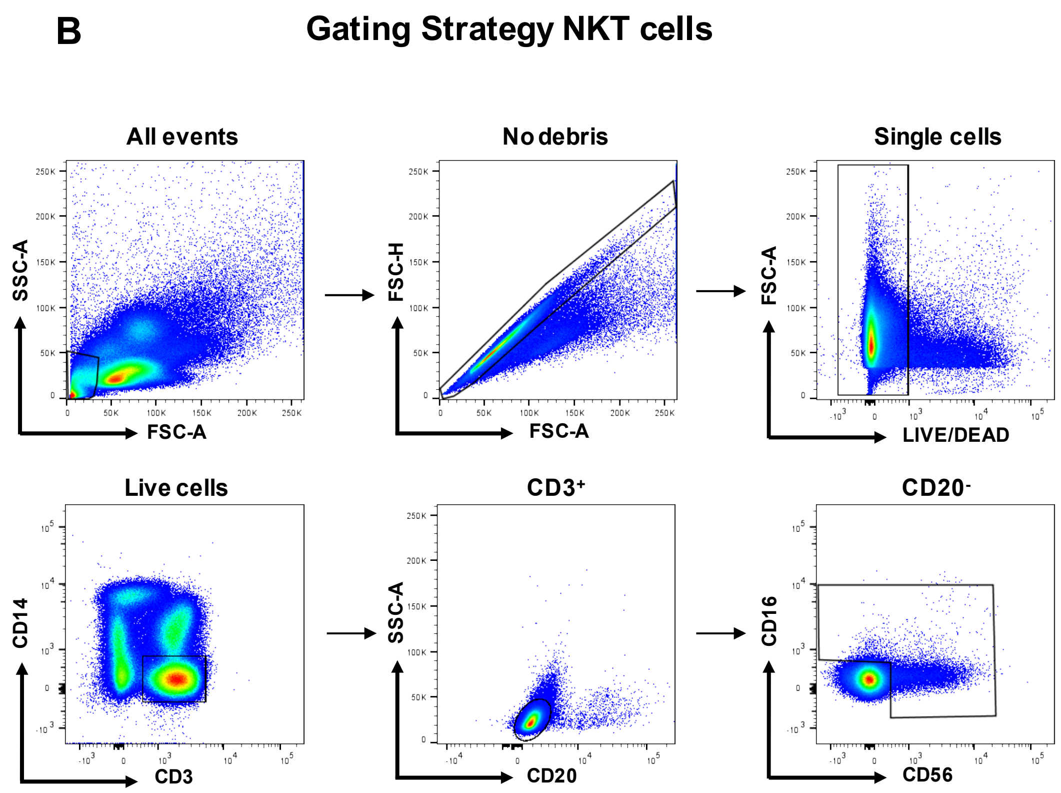
B
Gating Strategy NKT cells
All events
No debris
Single cells
SSC-A
FSC-A
FSC-H
FSC-A
FSC-A
LIVE/DEAD
Live cells
CD3+
CD20-
CD14
CD3
SSC-A
CD20
CD16
CD56

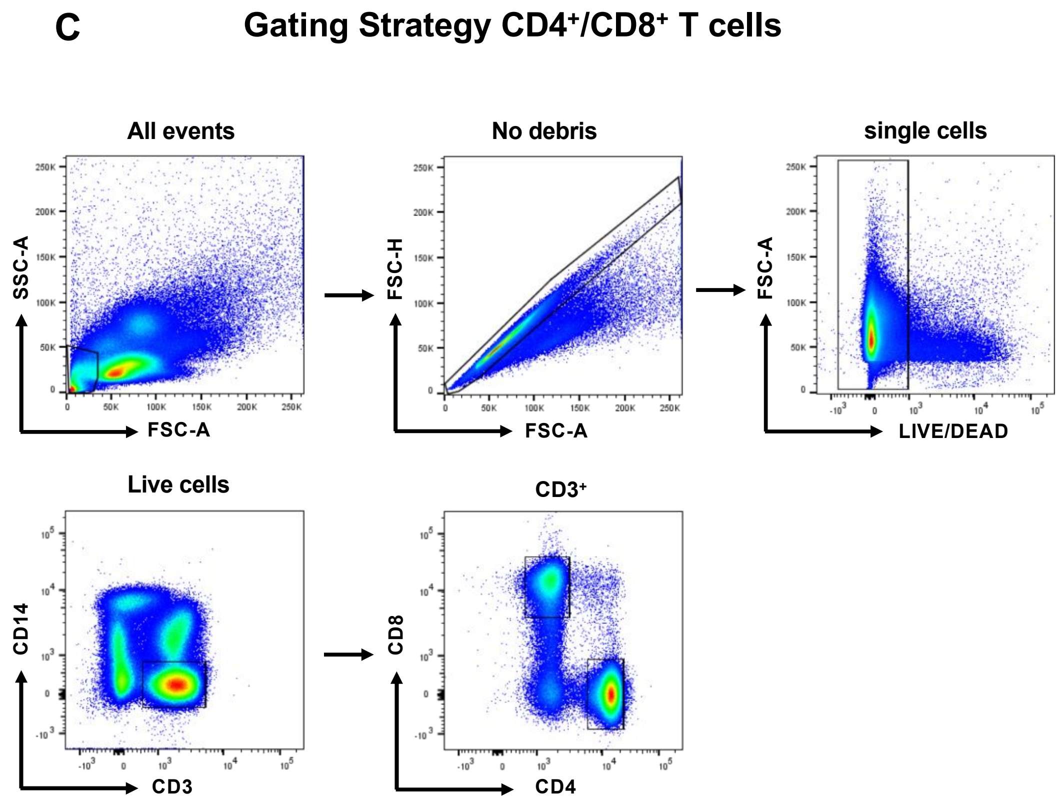
C
Gating Strategy CD4+/CD8+ T cells
All events
No debris
single cells
SSC-A
FSC-A
FSC-H
FSC-A
FSC-A
LIVE/DEAD
Live cells
CD3+
CD14
CD3
CD8
CD4

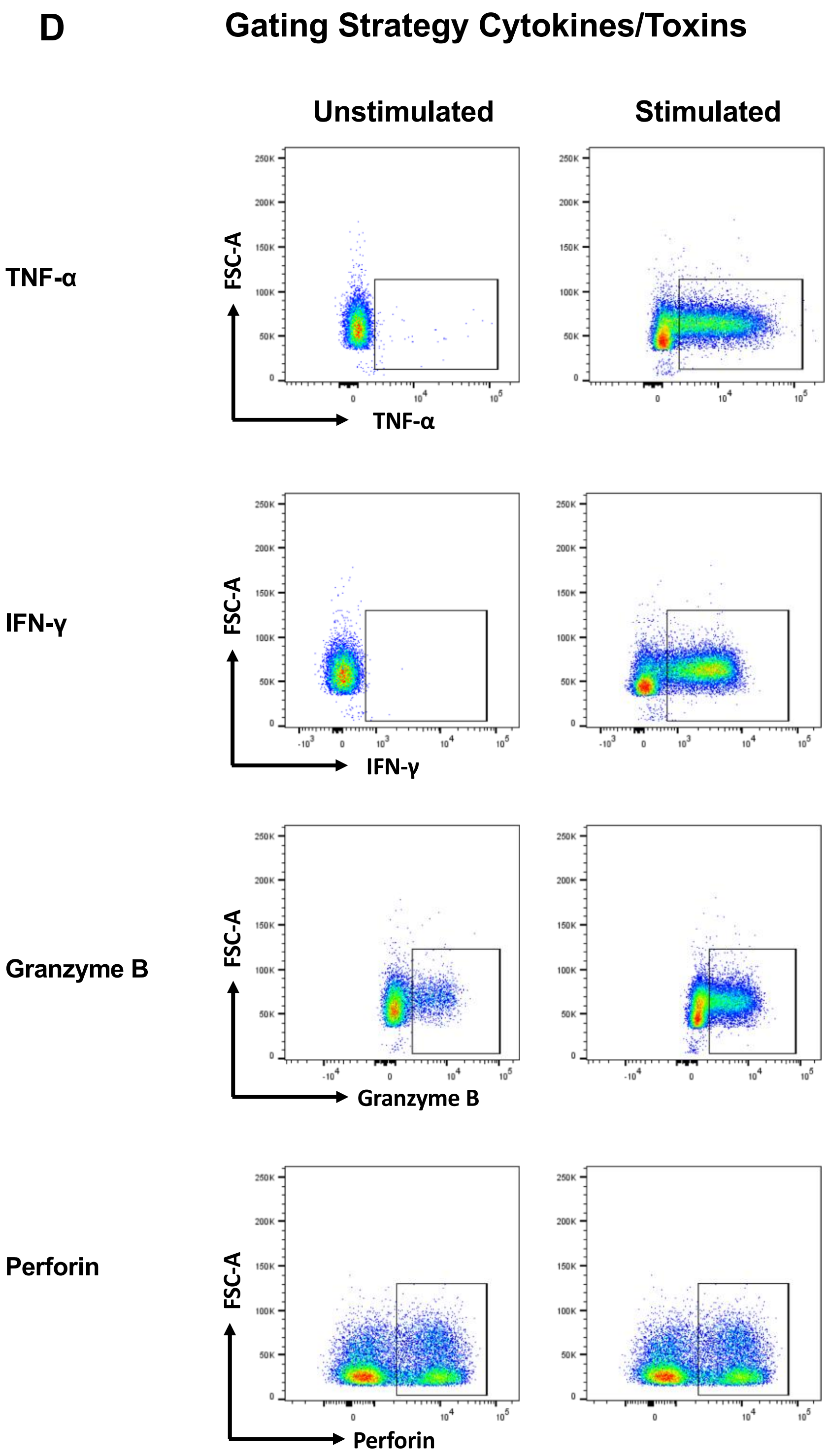

Supplementary Figure S9: Gating strategy to define NK cells (**A**), NKT cells (**B**) and CD4⁺/CD8⁺ T cells (**C**). The gates used to define TNF-α, IFN-γ, Granzyme B and perforin-producing cells are shown for representative examples of unstimulated and stimulated CD8⁺ T cells from a COVID-19 patient (**D**).
